# Cryo-electron microscopy reveals how acetogenins inhibit mitochondrial respiratory complex I

**DOI:** 10.1016/j.jbc.2022.101602

**Published:** 2022-01-19

**Authors:** Daniel N. Grba, James N. Blaza, Hannah R. Bridges, Ahmed-Noor A. Agip, Zhan Yin, Masatoshi Murai, Hideto Miyoshi, Judy Hirst

**Affiliations:** 1 MRC Mitochondrial Biology Unit, University of Cambridge, Cambridge, UK; 2 Division of Applied Life Sciences, Graduate School of Agriculture, Kyoto University, Kyoto, Japan

**Keywords:** complex I, cryo-electron microscopy, acetogenin, inhibitor-bound structure, binding site, CC_mask_, model–map correlation coefficient in a masked region, complex I, NADH:ubiquinone oxidoreductase, cryo-EM, single-particle cryomicroscopy, CTF, contrast transfer function, DDM, *n*-dodecyl-β-d-maltoside, DQ, decylubiquinone, FMN, flavin mononucleotide, FSC, Fourier shell correlation, IACS, Institute of Applied Cancer Science, OS-UQs, oversized ubiquinones, PEGylated, polyethylene glycol conjugated, THF, tetrahydrofuran

## Abstract

Mitochondrial complex I (NADH:ubiquinone oxidoreductase), a crucial enzyme in energy metabolism, captures the redox potential energy from NADH oxidation/ubiquinone reduction to create the proton motive force used to drive ATP synthesis in oxidative phosphorylation. High-resolution single-particle electron cryo-EM analyses have provided detailed structural knowledge of the catalytic machinery of complex I, but not of the molecular principles of its energy transduction mechanism. Although ubiquinone is considered to bind in a long channel at the interface of the membrane-embedded and hydrophilic domains, with channel residues likely involved in coupling substrate reduction to proton translocation, no structures with the channel fully occupied have yet been described. Here, we report the structure (determined by cryo-EM) of mouse complex I with a tight-binding natural product acetogenin inhibitor, which resembles the native substrate, bound along the full length of the expected ubiquinone-binding channel. Our structure reveals the mode of acetogenin binding and the molecular basis for structure–activity relationships within the acetogenin family. It also shows that acetogenins are such potent inhibitors because they are highly hydrophobic molecules that contain two specific hydrophilic moieties spaced to lock into two hydrophilic regions of the otherwise hydrophobic channel. The central hydrophilic section of the channel does not favor binding of the isoprenoid chain when the native substrate is fully bound but stabilizes the ubiquinone/ubiquinol headgroup as it transits to/from the active site. Therefore, the amphipathic nature of the channel supports both tight binding of the amphipathic inhibitor and rapid exchange of the ubiquinone/ubiquinol substrate and product.

NADH:ubiquinone oxidoreductase (complex I), located in the energy-transducing inner membrane of mitochondria, oxidizes NADH and reduces ubiquinone and couples the redox process to proton translocation across the membrane to generate the proton motive force that powers ATP synthesis and transport processes (Fig. 1). During catalysis, ubiquinone is reduced by electron transfer from iron–sulfur cluster N2, the terminal cluster in a chain that connects the active sites for NADH oxidation and ubiquinone reduction (1, 2, 3, 4, 5). The X-ray crystal structure of *Thermus thermophilus* complex I showed, for the first time, that this cluster is located at the junction between the enzyme’s hydrophilic and transmembrane domains. Cluster N2 sits approximately 30 Å above the membrane and is connected to it by a long channel, proposed to constitute the quinone-binding site (6). The same architecture has now been confirmed in complex I from a wide range of species including mammals, fungi, and plants (7, 8, 9, 10, 11, 12, 13). The long, narrow binding site forms the route for the redox-active quinone headgroup to access its catalytic active site adjacent to iron–sulfur cluster N2 and accommodates the length of its hydrophobic isoprenoid tail as it does so.

**Figure 1:**
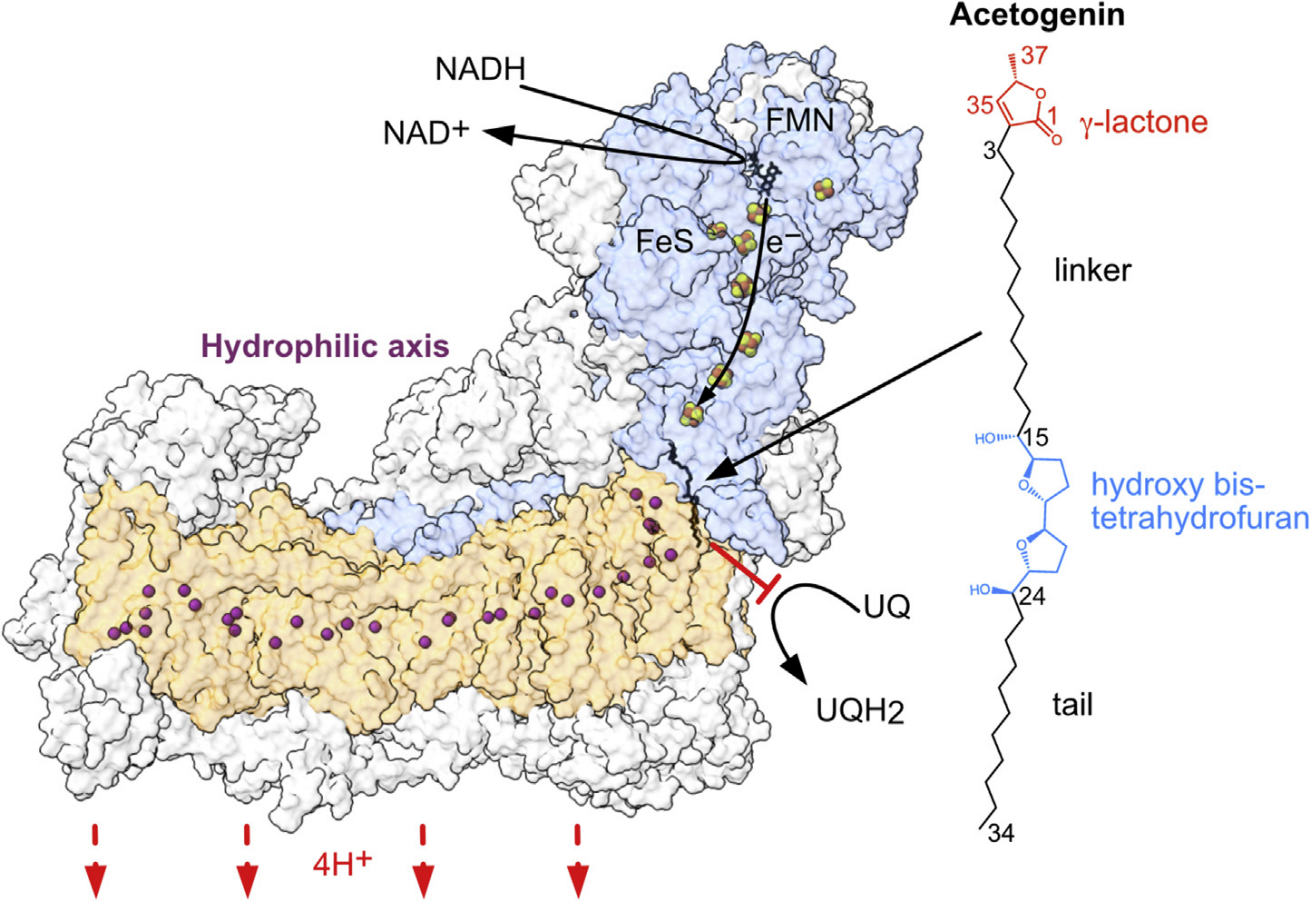
**The structure of acetogenin-bound mammalian complex I.** The amphipathic acetogenin inhibitor (compound **1**) occupies the ubiquinone (UQ)-binding site at the interface between the hydrophilic domain (*blue*) and the transmembrane domain (*orange*) core subunits. The iron–sulfur (FeS) clusters, responsible for electron relay from the bound flavin mononucleotide (FMN) following its reduction by NADH, are shown as *orange*–*yellow spheres*. Conserved and polar residues of the central hydrophilic axis that spans the transmembrane arm, implicated in the mechanism of proton translocation, are indicated by *purple spheres* at their respective Cα positions. The supernumerary subunits are shown in *gray*, with those obstructing the view of the core subunits removed for clarity.

The ubiquinone-binding site is formed by the membrane-domain subunit ND1 and two subunits in the hydrophilic domain, NDUFS2 and NDUFS7 (we use the human subunit nomenclature throughout). In the structurally defined ‘active’ resting state of the mammalian enzyme, the loop structures that form the binding site are clearly visible, and the ubiquinone-binding site is ‘substrate ready’. In contrast, local restructuring of several of these loops, plus an adjacent loop in membrane-bound subunit ND3, occurs concurrently with large-scale motions of the two domains to form the ‘deactive’ state of the mammalian complex—in which the ubiquinone-binding site is disrupted (7, 8, 9, 14). The deactive state forms spontaneously in the absence of substrates and can be reactivated upon their re-addition (15). The same loops may also move during catalysis, and structures that resemble the structurally assigned deactive state (8, 14) have been suggested recently to represent catalytic intermediates instead (16).

Once the long ubiquinone molecule is inserted fully into the channel, the two ketone groups on the ubiquinone headgroup moiety are thought to form hydrogen bonds with two residues of NDUFS2, H59 and Y108 (*Mus musculus* numbering used throughout), to allow rapid proton transfer to the nascent quinol following electron transfer from cluster N2 (6, 17, 18, 19, 20, 21, 22). The mechanism by which ubiquinone reduction is coupled to the relay of a signal to initiate proton translocation across the membrane is unknown. Proposed mechanisms include conformational adjustments triggered by sequential substrate reduction and compensatory stabilization of the intermediate/product (23, 24), an electrostatic signal from a conserved aspartate at the reduction site (20, 25), or the transit of ubiquinone/ubiquinol through a cluster of charged residues near a conserved stretch of glutamate residues at the top of the E-channel (6, 8, 10, 21, 26, 27). Structures of complex I are available with inhibitors bound in the ubiquinone-binding site, including piericidin A (6, 28, 29), rotenone (16) and IACS (Institute for Applied Cancer Science)-2858 (30) and smaller substrates such as decylubiquinone (6, 16, 28), but molecular details, even of how the ubiquinone headgroup is coordinated during its reduction, remain unconfirmed.

A group of natural product complex I inhibitors known as annonaceous acetogenins, derived from Annonaceae plants and shown to have antitumor effects (31), are much more hydrophobic than the inhibitors characterized in the aforementioned structural studies due to their substantial aliphatic carbon extensions and linker regions (Figs. 1 and 2). Their long, unbranched architecture, terminal γ-lactone group, and high hydrophobicity render them analogs to the native ubiquinone substrates of complex I (ubiquinones-9 and -10 in the mammalian system, with lengths of ∼40–45 Å) and thereby competitive inhibitors against it (32). On this basis, the chemical synthesis of orthologs of the natural product acetogenins, such as bullatacin, has been developed and has provided affinity-labeling data to probe the acetogenin-binding site in complex I, highlighting interaction sites with the core subunits ND1 and NDUFS2 located at the interface of the transmembrane and hydrophilic arms, respectively. Other modified orthologs have been used to elucidate the determinants of inhibition efficacy (33, 34, 35, 36, 37, 38, 39, 40) (Fig. 2). Here, we used a copurification procedure (29) to prepare acetogenin-bound mammalian complex I and determine its structure using single-particle electron cryomicroscopy (cryo-EM) to 3.4 Å resolution (Fig. 1). Our data provide the first experimental evidence for a molecule occupying the full length of the complex I binding channel and allow the molecular interpretation of structure–activity relationships within the acetogenin family.

**Figure 2:**
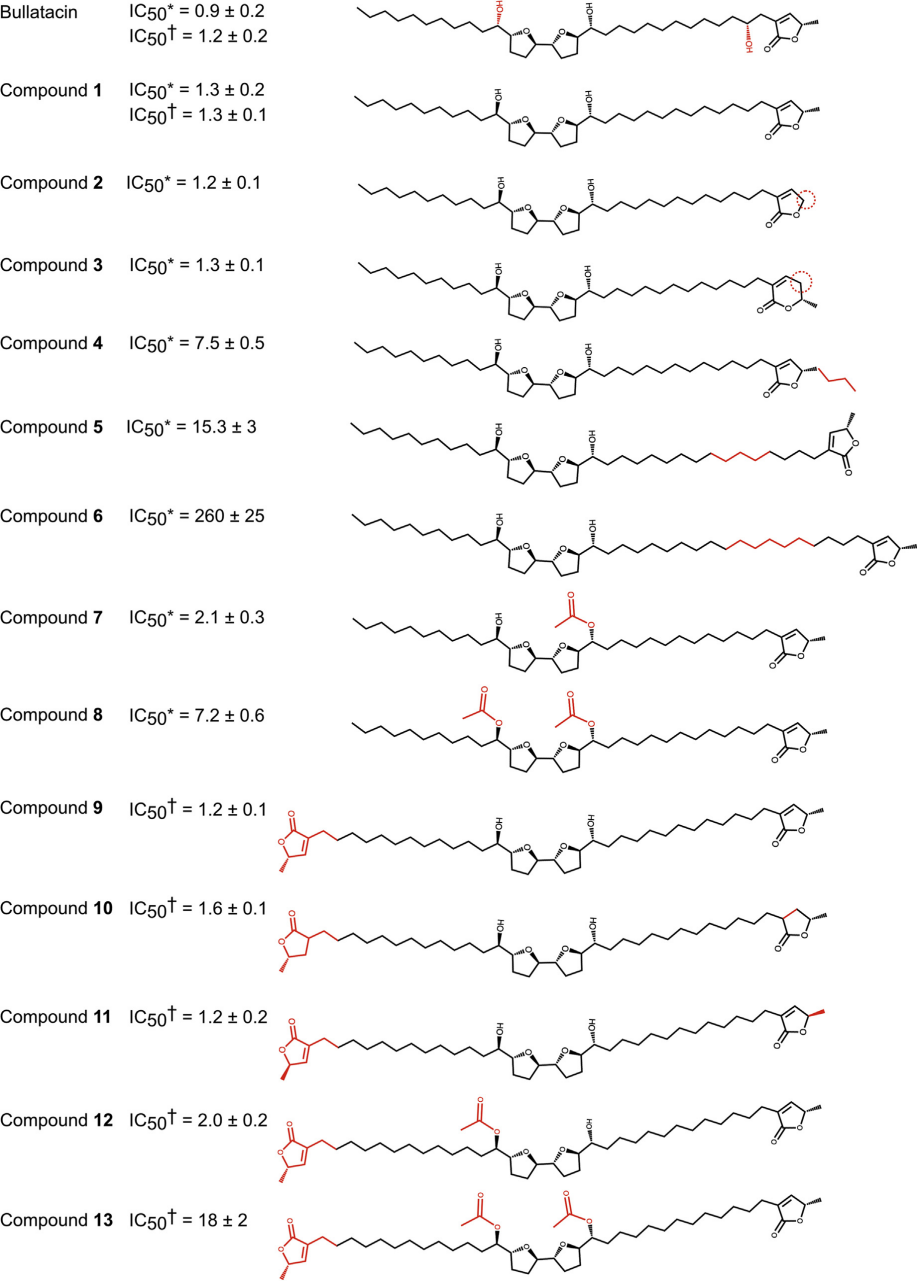
**Structure–activity relationships.** IC_50_ values (nM) for the natural product bullatacin and the various synthesized orthologs were assayed using bovine submitochondrial particles, with an internal bullatacin control for each study: ∗ (33), † (34). The chemical differences relative to compound **1** (studied here) are highlighted in *red*.

## Results

### Determination of the structure of acetogenin-bound mammalian complex I

To better understand the mode of acetogenin binding to complex I, the inhibitor-bound enzyme was prepared for cryo-EM analysis. The acetogenin ortholog inhibitor selected for study here is compound **1**; it contains a γ-lactone group, a 12-carbon aliphatic chain (linker) that connects the γ-lactone to a central hydroxylated bis-tetrahydrofuran (THF) group, and a final 10-carbon aliphatic extension (tail) (Figs. 1 and 2). Compound **1** exhibits potent complex I inhibition, comparable to the closely related natural product acetogenin bullatacin (34, 41). The highly hydrophobic compound **1** (calculated log*P* = 9.31) was added mid-way through the purification of complex I from mouse heart mitochondria, without the inclusion of complex I-damaging solvents. Furthermore, substrates (NADH and decylubiquinone, DQ) were added alongside to stimulate complex I turnover, along with an alcohol dehydrogenase and an alternative oxidase for substrate regeneration, to ensure exposure of the high-affinity binding site that may only be present in specific intermediates. Excess compound **1**, substrates, and regenerating enzymes were then removed by size-exclusion chromatography to eliminate nonspecifically bound inhibitor molecules. To estimate the proportion of inhibitor-bound enzyme present, the NADH:DQ assay was used, with low DQ concentration to minimize displacement of the inhibitor, in the presence of the alternative oxidase to re-oxidize quinol. Comparison of initial rates with a control sample, prepared identically but without addition of compound **1**, suggested that 77 ± 5% of the complex I molecules present contained the inhibitor.

**Figure 3:**
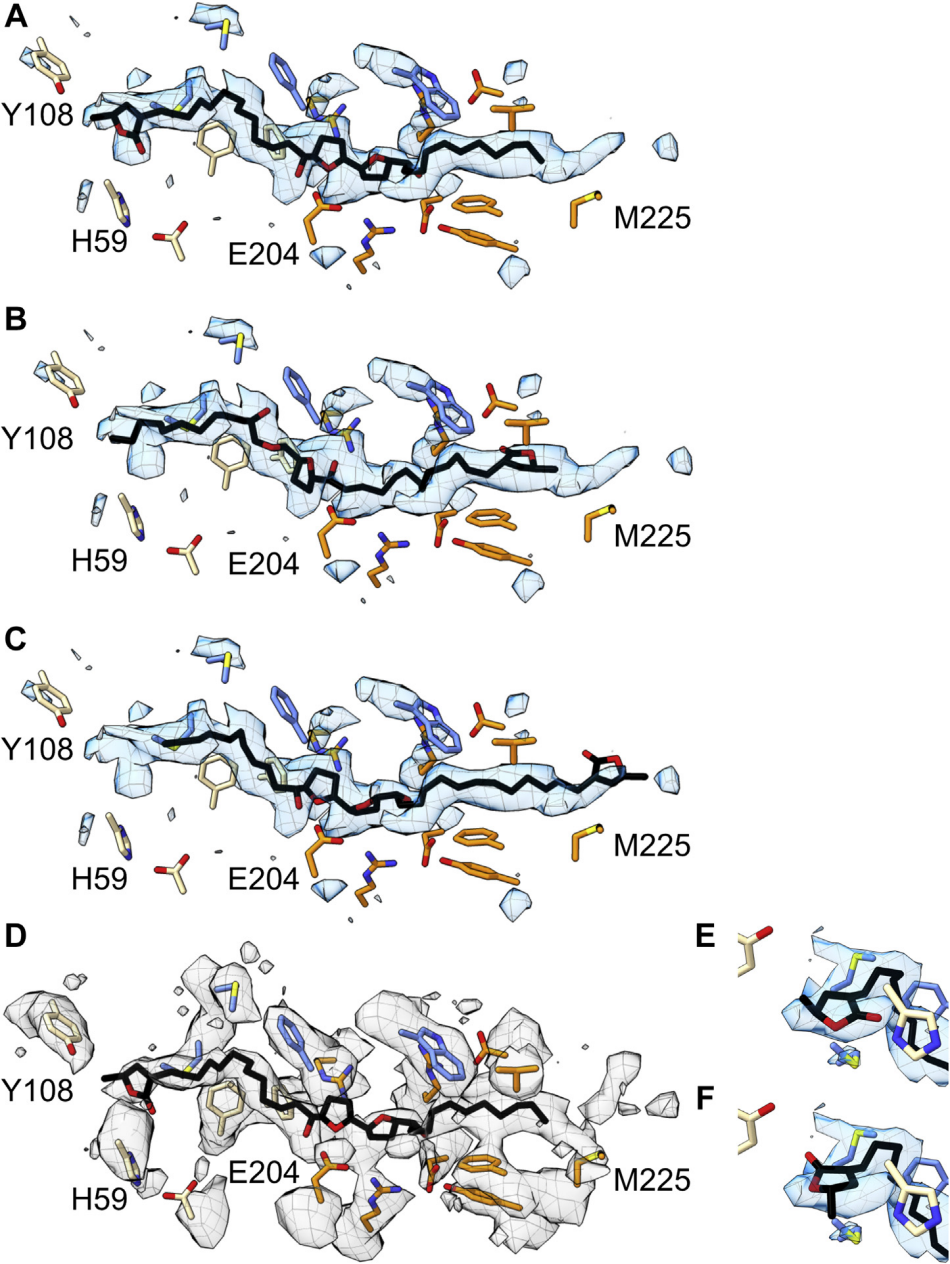
**The cryo-EM density for the proposed bound acetogenin ortholog compound 1.***A*–*C*, the map–model difference map (*blue mesh*, ChimeraX threshold 13.00), generated with the *phenix.real_space_diff_map* command with a compound **1**-free final protein model and locally sharpened consensus map and (*D*) the consensus locally sharpened map (*grey mesh*, ChimeraX threshold 0.03). The threshold was adjusted to best match the proposed acetogenin feature in the two maps. The compound **1** molecule is in *black stick*. *A*, the γ-lactone-first conformation (CC_mask_ = 0.724); *B*, the aliphatic chain-first conformation fit from deepest part of the channel, and (CC_mask_ = 0.674) (*C*) the aliphatic chain-first conformation fit to match the bis-tetrahydrofuran position of that in *A* (CC_mask_ 0.668). NDUFS2 residues are indicated in *wheat*; NDUFS7 in *blue*; and ND1 in *orange*. *D*, the final modeled position of compound **1** in the consensus map (CCmask = 0.654). *E*, the position of the final modeled γ-lactone and (*F*) the flipped orientation with CC_mask_ values (difference map) of 0.724 and 0.668, respectively. Cryo-EM, single-particle electron cryomicroscopy.

The inhibited sample was plunge-frozen onto PEGylated gold cryo-EM grids (14, 42, 43). The grid selected for data collection contained a good ice gradient and well-distributed particles (Fig. S1), and 1286 micrographs were obtained. Data were processed in RELION-3.1 (Fig. S2), resulting in a class of 16,440 particles with clear complex I features. The initial consensus refinement contained a discontinuous feature in the expected ubiquinone-binding site, which is partially composed of two subunits (ND1 and NDUFS2) that have both been implicated in acetogenin binding (32, 37, 38, 39). To improve the feature definition, the particles were subjected to signal subtraction, followed by focused 3D classification (see Experimental procedures). This process resulted in a marginal separation of a major class and a final consensus map at 3.4 Å resolution, with continuous cryo-EM density in the predicted acetogenin/ubiquinone-binding site (Figs. S2 and S3). The map was of sufficient quality for a model containing 8181/8430 residues (97%) plus ligands and lipids (Tables S1 and S2 and Fig. S4). The model was used to generate a map–model difference map (44) to inspect unmodeled cryo-EM densities for bound compound **1** molecules. Suitable unmodeled map features were isolated and inspected manually (see Experimental procedures). Given the high hydrophobicity of compound **1**, only membrane-bound protein regions were explored. Unmodeled features were identified on the periphery of the transmembrane domain but determined to be mobile lipid/detergent-based features that lacked sufficient continuous definition for modeling. Only one continuous difference density feature that resembled compound **1** was identified, contained within the ubiquinone-binding channel. It extends from the proposed ubiquinone ligands of H59 and Y108 of NDUFS2, passing near the acidic TMH5–6 loop of ND1, and ending near the membrane entrance to ND1, adjacent to ND1-M225 (Fig. 3) and accommodates an elongated conformation. However, given the symmetrical nature of compound **1**, multiple poses were explored in order to deduce the final pose: comparing their fit to the cryo-EM density, utilizing the model–map correlation coefficient in a masked region (CC_mask_) tool (45) to inspect local regions of correlation between the map and model, and evaluating the candidate protein–compound **1** interactions (Fig. 3). Although we cannot exclude a mixture of binding poses being present, we propose that the dominant state is the one in which the γ-lactone enters first and deepest into the channel. This gave the best fit to the cryo-EM density (CC_mask_ = 0.72 *versus* 0.67 and 0.68, Fig. 3) and best matched chemically favorable protein–compound **1** environments along the binding channel. Similarly, we favor the γ-lactone carbonyl group pointing toward NDUFS2-H59, supported by weak map features indicative of their interaction and a higher CC_mask_ value (0.72 *versus* 0.67); in the ‘flipped’ pose, the carbonyl points toward NDUFS7-V67 and the methyl group moves out of the curved cryo-EM density (Fig. 3, *E* and *F*).

### Acetogenin ortholog compound 1 binds to the active resting state of complex I

Cryo-EM particle classifications suggested the presence of only one global class of intact complex I. A single class is typical for the mouse enzyme (8, 29), whereas analogous analyses of the bovine and ovine enzymes typically comprise two (or more) major classes that differ globally in the angles between the two main domains (7, 8, 9, 16), which we attribute to the so-called ‘active’ and ‘deactive’ states. Comparison with published maps for the active (EMDB 11377) and deactive (EMDB 11810) states of the mouse enzyme, low-pass filtered to 3.4-Å resolution, indicated the compound **1**-bound complex (ChimeraX threshold 0.045) is in the active state (map correlation values 0.97 and 0.81 at ChimeraX thresholds 0.025 and 0.08, respectively). Differences between the active and deactive states also manifest in the local conformations of certain protein regions. In the active state, ND6-TMH3 (ND6 transmembrane helix 3) is completely α helical, and loops in NDUFS2, NDUFS7, and ND1 (TMH5–6) form an ordered binding site for ubiquinone. In contrast, the deactive state contains a π bulge in ND6-TMH3, and the aforementioned loops, plus the ND3-TMH1–2 loop, become less ordered, adding flexibility to the junction of the hydrophilic and transmembrane domains and likely promoting the altered global conformation (7, 8, 9, 14). The compound **1**-bound complex exhibits all the local hallmarks of the active state (Fig. S5). This result is consistent with introduction of compound **1** under turnover conditions, which stimulate the activation of any deactive enzyme present (14, 15). Indeed, given its chemical similarity with the ubiquinone substrate (terminal polar ring and hydrophobic tail), compound **1** binding may mimic substrate-induced activation (14, 46).

### Mode of acetogenin binding

**Figure 4:**
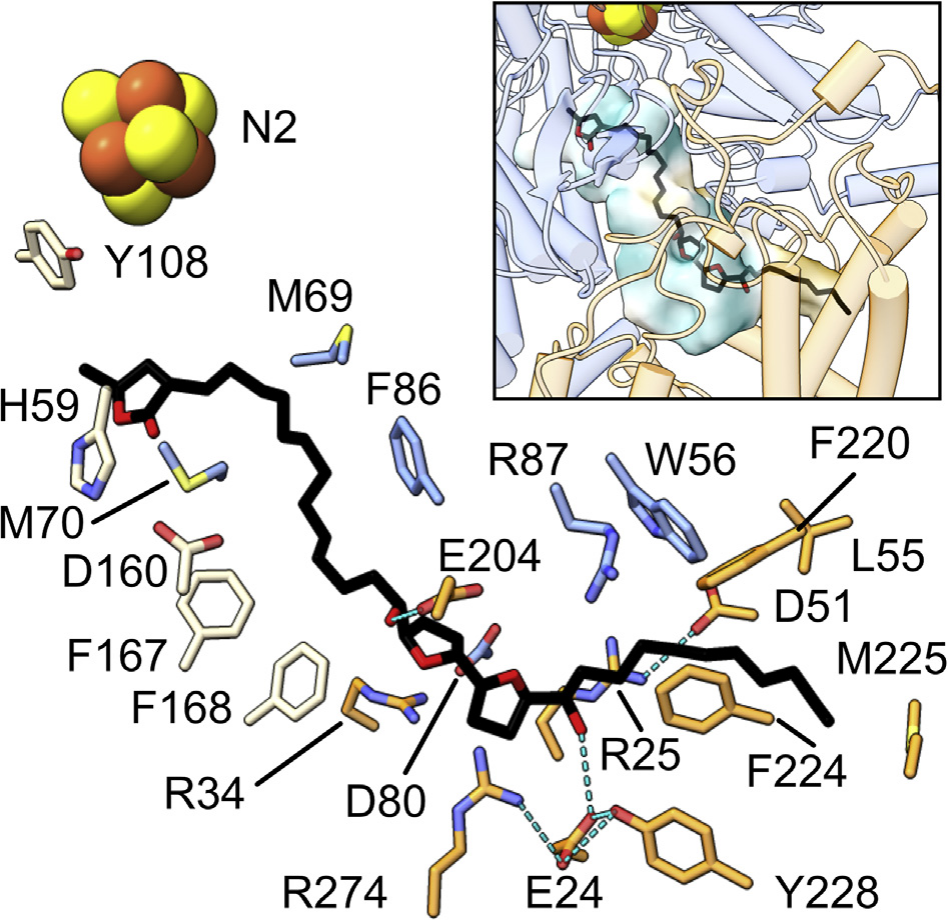
**The compound 1-binding site.** The modeled compound **1** (*black*) sits at the interface of the hydrophilic (*blue*) and transmembrane (*orange*) core domains (see *inset*), between residues of subunits ND1 (*orange*), NDUFS2 (*wheat*), and NDUFS7 (*blue*). The *inset* also shows the surface of the binding site cavity detected with a solvent probe (73), colored according to the hydrophilicity (*teal*)/hydrophobicity (*gold*) of the surrounding residues using ChimeraX. See also Fig. S6. ND1-TMH4 residues 128 to 134 are removed for clarity. *Blue dashed lines* indicate detected hydrogen bond interactions: ND1-E204 to compound **1**-C15-hydroxyl, ND1 R274–E24–Y228, R25–D51, and ND1-E24 to compound **1**-C24-hydroxyl.

**Figure 5:**
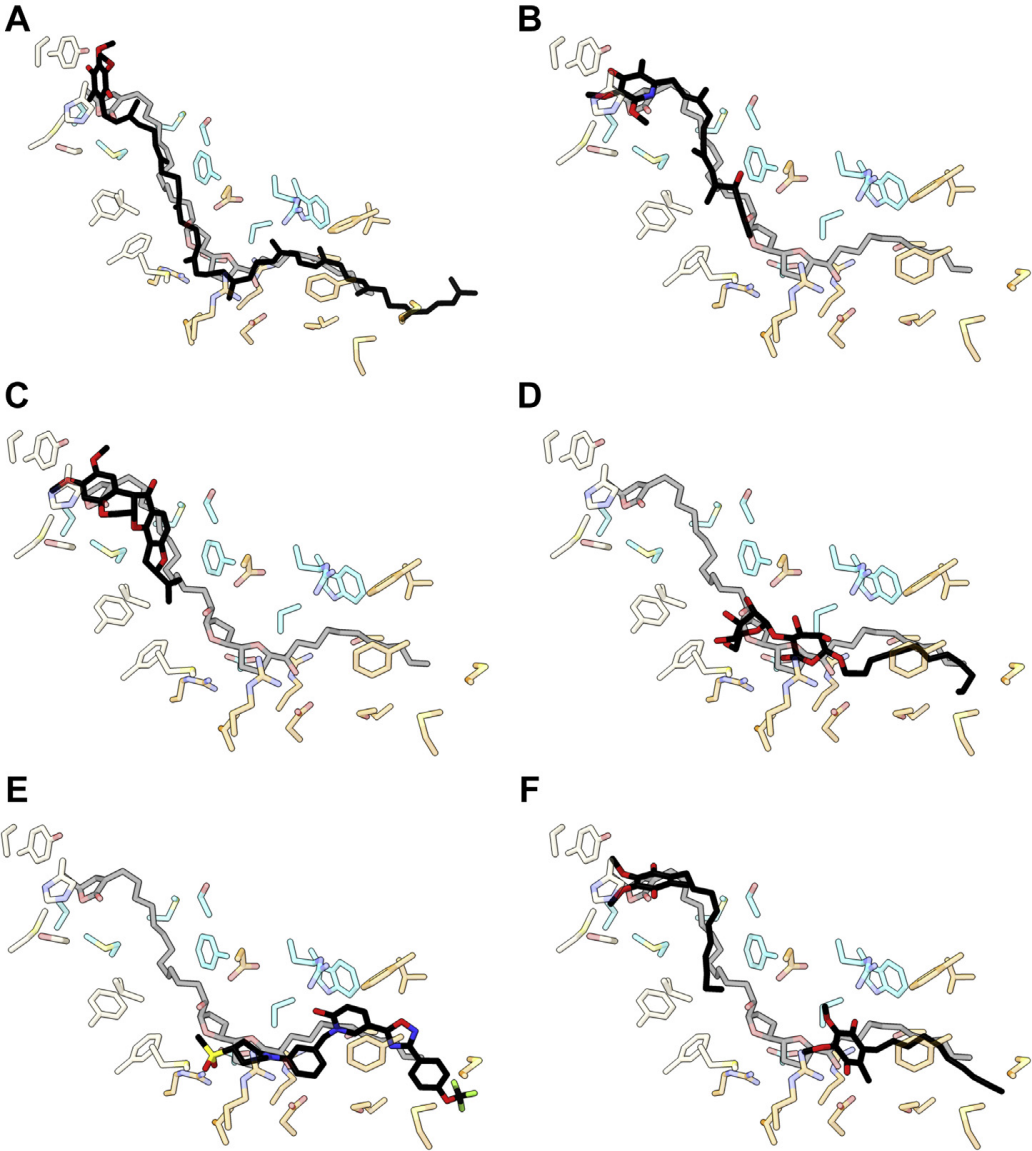
**Occupancy of the ubiquinone-binding site.** The bound conformation (*solid stick*) of (*A*) the simulation-predicted ubiquinone-10 as a native reference (26), the canonical complex I inhibitors (*B*) piericidin A (PDB 6ZTQ), (*C*) rotenone (PDB 6ZKK), (*D*) the detergent DDM (*n*-dodecyl-β-d-maltoside) (PDB 6YJ4), (*E*) the inhibitor IACS-2858 (PDB 7B93), and (*F*) the short-tail substrate decylubiquinone (PDB 6ZKC). The transparent residues and compound **1** reference are those obtained in this study, with the respective structures aligned *via* ND1, NDUFS2, and NDUFS7 subunits for the comparison.

The final model shows the ∼35 Å-long compound **1** molecule binds and occupies the expected ubiquinone-binding site entirely, spanning the full length of the channel at the transmembrane–hydrophilic domain interface formed by subunits ND1, NDUFS2, and NDUFS7 (Fig. 4). The properties of the residues that form the site produce an amphipathic tunnel with a central strongly polar and charged section flanked by hydrophobic distal regions (Fig. S6). The γ-lactone and hydroxylated bis-THF sit at the polar regions, with the linker and tail interfacing with the hydrophobic regions. This matches the amphipathic properties of the inhibitor with those of the channel and supports the γ-lactone-first pose modeled. The binding pose of the long, unbranched compound **1** tracks the same path proposed for ubiquinone-10 in simulations of its binding (Fig. 5*A*) (20, 21, 26, 27, 47).

The γ-lactone group enters the deepest into the channel from the membrane entry point, binding near to the terminal N2 cluster. It occupies a position close to (<4 Å) NDUFS2-H59 and forms hydrophobic packing contacts with NDUFS7-M70 and NDUFS2-Y108. H59 is modeled within hydrogen bonding distance of NDUFS2-D160, but not in a hydrogen bonding orientation to it.

Comparisons with cryo-EM structures of the piericidin A- and rotenone-bound mammalian enzymes (16, 29) show both inhibitors interacting with the same region as the γ-lactone and its linker (Fig. 5, *B* and *C*). All three inhibitors interact with NDUFS2-Y108; piericidin A and rotenone through hydrogen bonds to a ketone group and two methoxy groups, respectively, and compound **1** through a hydrophobic packing interaction. NDUFS7-M70 contributes hydrophobic packing interactions in all three cases—the aliphatic linker of compound **1**, the second isoprenoid unit of piericidin A, or the propylene-substituted ring of rotenone, respectively. NDUFS2-H59 is close enough to stably interact with both piericidin A and rotenone, but too far from compound **1**, for which the γ-lactone group is smaller. Attempts to reorientate the lactone ring to bring its oxygen atoms into hydrogen bonding positions with the protein residues while retaining it within the cryo-EM density were unsuccessful. Although the results suggest that the polar lactone ring does not form stable hydrogen bonds with nearby candidate residues, the cryo-EM density for the H59 side chain is less well defined than for the surrounding protein suggesting it adopts multiple rotamer conformations that may distribute it between interactions with either the γ-lactone or NDUFS2-D160. Indeed, the presence of compound **1** has promoted the movement of the NDUFS2-β1–β2 loop, and H59 within it, closer to NDUFS2-D160 than in the inhibitor-free mouse enzyme, similar to the movement in the piericidin A-bound structure (29).

The hydroxylated bis-THF group is located at the domain interface, where the compound **1** molecule crosses over from the membrane-bound ND1 subunit to the hydrophilic-domain NDUFS2 and NDUFS7 subunits (Figs. 1 and 4). This position is qualitatively similar to its predicted position at the membrane–solvent interface in molecular dynamic simulations of its behavior in a lipid bilayer (48). Photoaffinity experiments using labels on the hydroxylated bis-THF group suggested that it binds adjacent to a peptide that includes TMHs 5 and 6 and the TMH5–6 loop of ND1 (37); our model positions the hydroxylated bis-THF group directly opposite the gap between the two TMHs, with the C15 hydroxyl forming a hydrogen bond to E204 on the TMH5–6 loop, agreeing well with the labelling data. The only other hydrogen bond detected between compound **1** and the protein is between the C24 hydroxyl and the side chain of ND1-E24, which in turn forms a network with R274 and Y228 of ND1 (Fig. 4). Weak cryo-EM density features indicate, but not at a resolution to warrant modeling, water molecules between compound **1** and the protein, including one positioned to mediate hydrogen-bonding interactions between the side chain of ND1-E204 and the C15 hydroxyl (Fig. S7).

This bis-THF–occupied section of the ubiquinone-binding channel has been suggested to form a local energy minimum in simulations of ubiquinone transit (21, 27, 47) and has also been observed to be occupied by amphipathic molecules such as the detergent *n*-dodecyl-β-d-maltoside (DDM) (11), the IACS-2858 inhibitor (30), and the short-chain substrate DQ (16) in recent structures (Fig. 5, *D*–*F*). With each molecule, hydrophilic groups overlap the site of the compound **1** hydroxy bis-THF group: the two sugar rings of DDM and the terminal sulfone group and piperidine ring of IACS-2858 occupy the same positions as the two THF rings, and the DQ is located with a methoxy group and a benzoquinone carbonyl overlapping the distal THF and its hydroxyl group. The DDM-bound structure, with sufficient resolution (2.7 Å) to model water molecules, revealed just one direct contact between the protein and the ligand but eight contacts mediated by water molecules. It is thus likely that water-mediated contacts also dominate in contacts between the bis-THF group and the protein.

The bis-THF–occupied site is also of interest because it contains a highly polar selection of residues in a predominantly hydrophobic channel, in particular a set of acidic residues in subunit ND1. Of these, E227 creates a restriction point between the ubiquinone-binding cavity and a cavity inside the TMH 3 to 6 bundle of ND1 (11). Although caution should be taken when interpreting the modeled positions of acidic side chains as their densities are typically poorly defined (49), our map shows good definition for E227 and suggests it is stabilized by nearby ND1-R195 and a water molecule between E227 and Y228 (Fig. S7). No protein hydrogen bonding partners for E227 are present. Despite ambiguity in precise acidic side chain positions, our analysis highlights the potential role of this highly acidic region in regulating the connectivity between the ubiquinone-binding cavity and the E-channel, consistent with proposals from simulations (5, 20).

### Acetogenin structure–affinity relationships

**Figure 6:**
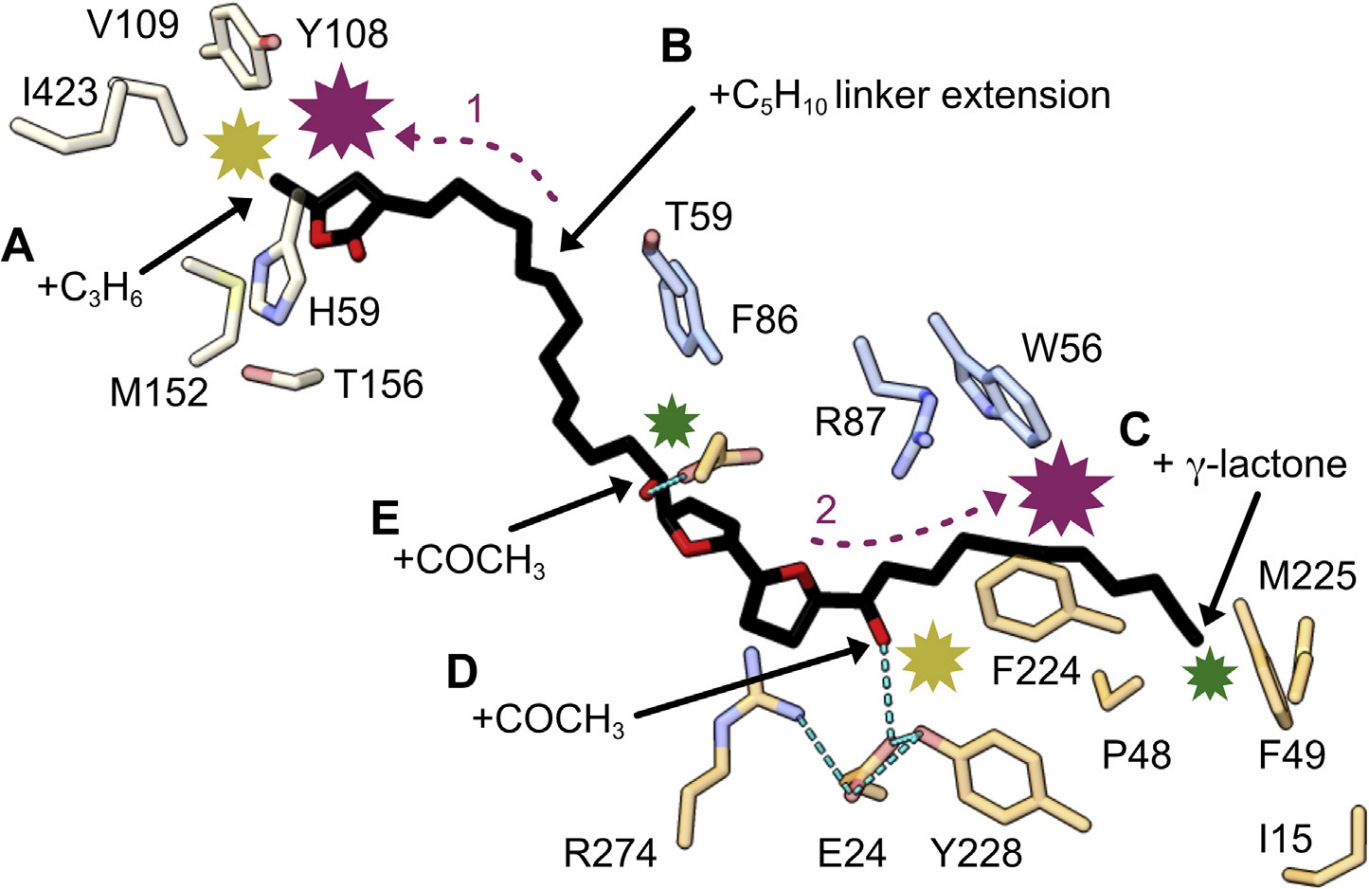
**Chemical modifications of the compound 1 molecule and structure-based rationalization of their altered inhibitory potency.** Key groups of compound **1** were explored with alternately synthesized compounds (33, 34), testing their inhibition efficacy through IC_50_ values (nM) in assays on bovine submitochondrial particles (Fig. 2). The central compound **1** (IC_50_ = 1.3 nM) is shown from the same viewpoint as Figure 4. Modifications and their effects on binding-site interactions were predicted though modeling these compounds in the cryo-EM density and exploring the nearby interactions: The prediction model atoms for *A*, *C*, *D*, and *E* were superimposed on those of the template compound **1** (except for extensions); the prediction model for *B* was generated by overlaying the lactone rings or the bis-THF regions. Example compounds are (*A*) compound **4** (C_3_H_6_ extension of the γ-lactone methyl group, IC_50_ = 7.4 nM); (*B*) compound **6** (C_5_H_10_ linker extension, IC_50_ = 260 nM); (*C*) compound **9** (addition of a C34 γ-lactone, IC_50_ = 1.2 nM); (*D*) compound **8**, (acetylation of the C24 hydroxyl group, IC_50_ = 7.2 nM); (*E*) compound **7** (acetylation of the C15 hydroxyl group, IC_50_ = 2.1 nM). Contact sites between the compound extensions (*black arrows*) and the protein residues (ND1, *orange*; NDUFS2, *wheat*; NDUFS7, *blue*) are indicated by starbursts for regions of interaction (<4 Å) and are sized and colored by relative degree of change on the IC_50_ value of the parent compound (*green*, mild; *yellow*, moderate; *purple*, severe). *Purple arrows* and *numbers* indicate the shift and respective clashes of the chemical groups when the linker extension of compound **6** is introduced: (1) held at the bis-THF site and the γ-lactone clashes or (2) held at the γ-lactone site the bis-THF clashes. THF, tetrahydrofuran.

The acetogenin family has been dissected and varied through alternate chemical syntheses in order to identify the key features that confer its potent inhibitory properties. Here, the IC_50_ values measured using assays with bovine submitochondrial particles (33, 34, 35), which denote the efficacy of inhibition, are rationalized for a range of the compounds tested (Fig. 2) using our structural data. Unless noted, all the residues we discuss are conserved in both the mouse and bovine enzymes. Compounds with the same basic structure as compound **1** (Fig. 1), but that are extended or truncated in different regions (Fig. 2), were positioned into our cryo-EM compound **1** density to explore the interactions that may explain their different IC_50_ values. The compound structures were generated using the Lidia tool and manually refined into the cryo-EM density using Coot (see Experimental procedures). Equivalent atom regions were matched to the modeled compound **1** (*e.g.*, the γ-lactone groups were overlaid), and then the extensions were positioned to optimize favorable interactions and reduce steric clashes. The surrounding protein was left unchanged, and the resulting suggested binding modes (Fig. 6) were evaluated.

The natural product bullatacin reference compound used in the IC_50_ structure–activity relationship studies is very similar to compound **1** used here except that C4 is hydroxylated (see Fig. 1 for the numbering systems used) and the stereochemistry at the C24 hydroxyl is different (Fig. 2). The close match of the IC_50_ values for bullatacin and the compound studied here shows that neither change substantially affects the binding interactions (41).

Adjustments were made to the lactone headgroup in compounds **2** (truncation of the R2 methyl to hydrogen), **3** (5- to 6-membered ring), and **4** (extension of the R2 methyl to *n*-butyl). Only the IC_50_ value of compound **4** was affected with a small increase to 7.5 nM, consistent with introduced steric clashes with NDUFS2-V109, M152, T156, I423, and V424 (Fig. 6*A*). In compounds **7** and **8**, the hydrophilicity of the two THF hydroxyl groups was explored by acetylation; this not only removes their hydrogen bonding capacity but also increases their size (33, 34). Compound **7** (Figs. 2 and 6*E*) is acetylated only on the C15 hydroxyl, whereas compound **8** is doubly acetylated (C15 and C24) (Figs. 2 and 6, *D* and *E*). Both compounds still show potent inhibition with IC_50_ values of 2.1 nM and 7.2 nM, respectively, compared to the 1.2 nM control. Although both hydroxyls form hydrogen bonds to protein residues, the data tentatively suggest the C24 acetylation to be more detrimental, perhaps due to its disruption of the binding site R274–E24–Y228 interaction or that of the additional stabilization by a water molecule linking it to NDUFS7-R87 (Fig. S7). The mobility (weaker cryo-EM density) of the E204 side chain that interacts with the C15 hydroxyl may better accommodate the acetyl group.

Compounds **9**, **11**, and **12** have lactone groups on both ends, producing a symmetrical molecule that can insert in both directions, and both maintain their high efficacy. When modeled into the current cryo-EM density map, the ∼7 Å extension, compared to the aliphatic chain of compound **1** studied, places the terminal lactone at the exit into the lipid environment forming packing interactions with I15 (L15 in bovine), P48, F49 (I49 in bovine), and M225 of ND1 (Fig. 6*C*). In addition, compounds **13** and **14** (Fig. 2) show that the effects of acetylation are preserved on the framework of the symmetrical molecule with a lactone on each end; the single acetylation (compound **13**) maintains its high affinity, and as expected, the double acetylation caused a marked increase, to 18 nM in this case.

The clearest result from these comparisons, however, is that compound **1** used in this study is excellently adapted to inhibit the complex I ubiquinone-binding tunnel because it contains two hydrophilic moieties spaced by hydrophobic linkers, in a configuration that matches the corresponding properties of the channel (Fig. S6), to create a ‘bivalent’ ligand. The requirement of a linker region between the bis-THF group and the γ-lactone group to elicit cooperativity in binding and therefore inhibitor potency was previously discussed (34). Not only does the linker increase the overall hydrophobicity and facilitate entry into the complex from the membrane, it also optimally places the hydrophilic groups in the hydrophilic patches of the complex I tunnel. The effects of the linker length were explored with compounds **5** and **6** (Fig. 2). The additional carbon atoms in the linker result in steric clashing at the γ-lactone region and surrounding protein (NDUFS2 H59 and Y108, see also Fig. 6*A*) when the bis-THF group is held in the position of the modeled compound **1**. When the position of the γ-lactone is held to be the same as for compound **1**, the hydroxy bis-THF group is displaced toward the channel entrance introducing a chemically unfavorable mismatch between the hydrophilic and hydrophobic environments of the binding channel and the amphipathic ligand (Fig. S6). Additionally, the steric strain at the position of ND1-F224 (Fig. 6*B*) in this conformation can be alleviated by further displacement of the compound into the membrane, freeing the substrate-binding channel. Alternatively, to avoid steric hinderance from the extension, this compound may also enter in the reverse direction (tail first).

## Discussion

The mechanism of ubiquinone oxidoreduction-coupled proton translocation in complex I is not known, despite high-resolution structures (11, 16). The coupling reaction likely involves the residues that compose the ubiquinone-binding site and those that lead from it toward the transmembrane domain. However, even the binding mode of ubiquinone is uncertain; it has only been predicted from simulations (20, 21, 27, 50) as no fully bound ubiquinone structure has been determined. Structures containing inhibitors, detergents, and smaller substrates have been described, but these molecules only occupy discrete subsites along the predicted channel (6, 10, 11, 16, 29, 30). Ubiquinone binding to complex I has also been explored using synthesized ubiquinone derivatives of varying lengths, with bulky groups (1-methoxy-2,6-di(3-methoxy-3-methyl-1-butynyl)benzene) added to the end of their tails—known as ‘oversized ubiquinones’ (OS-UQs). The bulky group is too large to pass through the entrance to the ubiquinone-binding channel from the membrane, and if the linking tail is not long enough, the ubiquinone headgroup is unable to reach its catalytic site at the end of the channel (51, 52). Bovine complex I reconstituted into proteoliposomes was only able to catalyze the inhibitor-sensitive reduction of OS-UQ8, which has a tail length equivalent to eight isoprenoid units, predicted by the structure to be just long enough. The inactivity of OS-UQ1–7, which are prevented from entering sufficiently far into the channel by the bulky group, is fully consistent with the proposed ubiquinone-binding channel. We note that the behavior of the amphiphilic OS-UQ1–3 differed between the complex I proteoliposomes and a native membrane system, and the structure of the detergent-solubilized, inhibitor-bound enzyme presented here offers no new insights into this differing behavior.

Here, an acetogenin compound that tightly binds and inhibits complex I was studied. Acetogenin molecules resemble the native ubiquinone-9/10 substrate, being highly hydrophobic with an extended conformation, and with a γ-lactone ring in the equivalent position of the ubiquinone headgroup (Figs. 1 and 2). We employed a copurification method (29) and cryo-EM to show that, uniquely among complex I inhibitors, the long compound **1** acetogenin binds the entire length of the predicted ubiquinone-binding channel of mammalian complex I.

Comparisons of the compound **1**-bound state with earlier uninhibited mouse structures (8, 29), which contained no identifiable species in their binding sites, highlight subtle differences in a set of residues in subunit ND1 at the hydrophilic kink region, including glutamates E202, E204, and E227, which have been implicated in proton transfer to the E-channel to initiate proton translocation (5, 20). The compound **1**-bound model here favors the conformation of these residues that restricts accessibility from the binding channel to the E-channel. As the central bis-THF moiety of the compound is hydrophilic and sits directly in the hydrophilic kink region of the binding tunnel that was shown to be a ‘checkpoint’ in ubiquinone transit (21, 27), it is interesting to speculate that the compound **1**-bound state mimics a state formed as the ubiquinone headgroup pauses in this region and primes the coupling mechanism.

Simulations of the central section of the channel have also suggested that its structure and local side chain orientations may respond to channel occupancy through the formation of a hydrogen bonding triad (ND1-R25 to NDUFS7-D80 to ND1-R34) when ubiquinone is fully bound but that the hydrogen bonds are weaker/more transient when the ubiquinone headgroup pauses at the central checkpoint during transit (53). In our compound **1**-bound structure, the central D80 has a poorly defined side chain, suggesting it is deprotonated or occupying multiple rotamer conformations through transient hydrogen bonds to R25 and R34 (Fig. 4), consistent with the hydrophilic bis-THF moiety mimicking a checkpoint state. A robust evaluation of the significance of these subtle changes must await a fully ubiquinone-bound structure, with the headgroup bound at the deepest position of the channel and the hydrophobic isoprenoid chain running through the checkpoint site. We note that, in a separate set of simulations, a further stable hydrogen bond between ND1-R25 and ND1-D51 was also proposed to be specific to the fully ubiquinone-bound state (27) but is observed in our checkpoint-like state.

Simulations of substrate binding by Teixeira and Arantes suggested that long-tailed ubiquinones that occupy the full length of the channel, such as ubiquinone-10, have much larger free energy cost for binding when compared to shorter analogs (27). With the headgroup bound in its active site, the hydrophobic isoprenoid units of the long-tail ubiquinones must bear the cost of displacing water molecules from the central polar region of the cavity (Figs. S6 and S7), whereas the tails of shorter species, such as ubiquinone-2, do not extend far enough. The cost is incurred as the ubiquinone headgroup moves from the checkpoint site—where the headgroup is bound and only the first couple of isoprenoid units occupy the channel entrance—to the active site. The penalty for hydrophobic ligand binding at the polar kink site suggests that the binding site may have evolved to decrease its affinity for long hydrophobic compounds in order to prevent turnover being limited by their rates of dissociation. This proposal is in agreement with kinetic data showing that catalytic efficiency increases with ubiquinone tail length, with ubiquinones 8 to 10 displaying the highest values (26). In contrast, the hydroxylated bis-THF group of the acetogenin inhibitors is stable, favorable even, in the checkpoint site, and so this family of long and hydrophobic inhibitors avoids the free energy binding penalty. Our observations combine to suggest that acetogenin class are such potent inhibitors because they contain two specific hydrophilic moieties, spaced out along their length, that locate and bind into two specific hydrophilic regions, equivalent to the predicted local energy minima zones of ubiquinone binding and transit through the channel.

## Experimental procedures

### Preparation of acetogenin compound 1-inhibited mouse complex I

Compound **1**-inhibited mouse complex I was prepared by adapting previous methods of inhibitor copurification with complex I (29), preparation of heart mitochondria (54) and purification of complex I (8). Briefly, hearts were obtained from C57BL/6 mice by cervical dislocation in accordance with the UK Animals (Scientific Procedures) Act, 1986 (PPL: 70/7538, approved by the local ethics committees of the MRC Laboratory of Molecular Biology and the University of Cambridge and by the UK Home Office) and the University of Cambridge Animal Welfare Policy. Hearts were excised, immersed in ice-cold storage buffer containing 10 mM Tris–HCl (pH 7.4 at 4 °C), 75 mM sucrose, 225 mM sorbitol, 1 mM EGTA, and 0.1% (w/v) fatty acid-free bovine serum albumin (Sigma-Aldrich), then cut into approximately 1-mm pieces, and washed with storage buffer. The tissue was homogenized in storage buffer (0.1 g ∙ ml^−1^) by 7 to 10 strokes of an IKA RW 20 homogenizer fitted with a Teflon pestle at ∼1400 rpm, then the homogenate was centrifuged (1000*g*, 5 min), and the supernatant recentrifuged (9000*g*, 10 min) to collect the crude mitochondria. The mitochondria were resuspended in 20 mM Tris–HCl (pH 7.4 at 4 °C), 1 mM EDTA, and 10% (v/v) glycerol to 10 to 20 mg protein ml^−1^ and frozen for storage. The typical time to obtain frozen mitochondria from the mouse was less than 2 h. After thawing, mitochondria were diluted to 5 mg protein mL^−1^ with resuspension buffer, then ruptured by three 5-s bursts of sonication (with 30 s intervals on ice), using a Q700 Sonicator (Qsonica) equipped with a microtip probe, at the 65% amplitude setting. The mitochondrial membranes were collected by centrifugation (75,000*g*, 1 h), resuspended to ∼5 mg protein ml^−1^, and frozen for storage.

All subsequent steps were performed at 4 °C unless stated otherwise. Starting from 4.3 mg protein ml^−1^ of membrane protein (3.4 ml), membranes were solubilized by drop-wise addition of 1% DDM (Glycon) along with 0.005% phenylmethylsulfonyl fluoride (w/v) and stirred for 30 min on ice before centrifugation (48,000*g*, 30 min). The supernatant was loaded onto two, in-tandem 1 ml Hi-Trap Q HP anion exchange columns (GE Healthcare) pre-equilibrated with buffer A [20 mM Tris–HCl (pH 7.14 at 20 °C), 1 mM EDTA, 0.1% DDM, 10% (v/v) ethylene glycol, 0.005% soybean asolectin (20% soy PC, Avanti) and 0.005% CHAPS (Calbiochem)]. The column was washed at 0.3 ml min^−1^ with buffer A for 0.3 ml, then with 20% buffer B (buffer A + 1 M NaCl) for 4 ml, and then complex I was eluted in 35% buffer B over 4 ml (8).

Complex I-containing fractions were pooled and concentrated to ∼250 μl using a 100 kDa MWCO spin column (Amicon Ultra-15), and the following were then added to induce and maintain complex I turnover (29): 200 μM NADH, 0.15% asolectin (20% soy PC, Avanti), 0.15% CHAPS (Calbiochem), and 6 μM decylubiquinone; 120 μg ml^−1^ alcohol dehydrogenase from *Saccharomyces cerevisiae* (Sigma) and 1% ethanol to regenerate the NADH from NAD^+^; 100 μg ml^−1^ alternative oxidase from *Trypanosoma brucei brucei* (AOX) to regenerate the ubiquinone from ubiquinol; and 6.7 KU ml^−1^ catalase from *Corynebacterium glutamicum* (Sigma) and 267 U ml^−1^ superoxide dismutase from bovine erythrocytes (Sigma), both to minimize oxidative damage.

After ∼5 min at 4 °C incubation, 20 μl of the mixture was removed as a control, and the remaining sample was added to glass vials containing enough dried compound **1**, from an ethanol stock, to give a final 200 μM of compound **1**, resuspended with gentle pipetting. The control portion was loaded first onto a Superose 6 Increase 3.2/300 size-exclusion chromatography column using an ÅKTA Micro (both GE Healthcare), eluting complex I at ∼1.2 ml [20 mM Tris–Cl (pH 7.14 at 20 °C), 150 mM NaCl and 0.05% (w/v) DDM]. The compound **1**-inhibited sample was then loaded, and compound **1**-bound complex I was eluted at ∼1.2 ml [20 mM Tris–Cl (pH 7.14 at 20 °C), 150 mM NaCl and 0.05% (w/v) DDM]. The inhibition of initial rates (first 25 s) of 77 ± 5% (SD, *n* = 3 technical repeats) was measured against the control sample using an assay containing 20 mM Tris–HCl (pH 7.5 at 20 °C), 0.15% asolectin (20% soy PC, Avanti), and 0.15% CHAPS, 5 μM DQ, and 0.5 μg ml^−1^ AOX, with catalysis initiated by 200 μM NADH and monitored at 340 to 380 nm (ε = 4.81 mM^−1^ cm^−1^). The low DQ concentration was used to minimize competition with compound **1**. The specific activity of the control [measured in 0.15% asolectin (20 % soy PC), 0.15% CHAPS, and 200 μM DQ] was 9.6 ± 0.4 μmol min^−1^ mg^−1^.

Compound **1** used for the preparation of the acetogenin-inhibited complex I was synthesized by the method described previously (33).

### Cryo-EM grid preparation, data acquisition, and processing

UltrAuFoil gold grids (0.6/1, Quantifoil Micro Tools GmbH) were glow discharged (20 mA, 90 s), incubated in a solution of 5 mM 11-mercaptoundecyl hexaethyleneglycol (PEG thiol, SensoPath Technologies) in ethanol for 2 days in an anaerobic glovebox, then washed with ethanol and air-dried just before use (14, 42, 43). Using an FEI Vitrobot Mark IV, 2.5 μl of acetogenin-inhibited complex I solution (4.6 mg ∙ ml^−1^) was applied to each grid at 4 °C in 100% relative humidity and blotted for 14 s at force setting –10, before the grid was frozen by plunging it into liquid ethane.

The data were collected at the Electron Bio-Imaging Centre at the Diamond Light Source (Harwell Science and Innovation Campus) using a 300 keV FEI Titan Krios. A Gatan K2 summit camera with energy filter (20 eV) was used in counting mode. A single grid (2-day PEG thiol incubation, –10 blotting force, 14 s blotting time, and 4.6 mg ml^–1^ complex I) was used, and 1286 micrograph images were collected using EPU software over 2 days. A total dose of 50 electrons/Å^2^ was used over 50 frames with an exposure time of 10 s, focusing every 10 μm. The defocus range was –1.5 to –2.7 μm, and the calibrated pixel size was 1.043 Å. The session reference is EM18074-1.

Data were processed using RELION-3.1. Beam-induced motion was corrected using MotionCor2 (55) with and without dose weighting. Per-micrograph contrast transfer function (CTF) correction was performed with GCTF (56) on nondose-weighted micrographs. A subset of 7000 particles were manually picked and subjected to 2D classification to generate 2D templates for subsequent autopicking where 114,209 particles were selected. These particles were re-extracted from CTF-estimated micrographs at an amplitude contrast value of 0.2 as this provided better 2D classification over 100 classes. From these 100 classes, 44,782 particles were selected and put forward to 3D classification over four classes. Just 16,440 particles were retained from a single good 3D class that resembled complex I. Higher angular sampling did not split this class of good complex I particles into two global states, as was previously observed for the mammalian enzyme (7, 8, 9). 3D autorefinement, using solvent-flattened Fourier shell correlation (FSC), was performed with subsequent per-particle CTF refinement, including higher-order correction (57) and Bayesian polishing (58, 59).

Focused classification of signal-subtracted particles focused around the predicted compound **1**-binding site was performed to reduce heterogeneity at this position and improve definition of the acetogenin cryo-EM density. The signal-subtracted particles were generated using a mask of 7-Å protein radius around the compound **1**-binding site, based on a preliminary model. The 3D volume of this region was generated and low-pass filtered to 15 Å using UCSF Chimera-1.13 (60), and signal-subtraction mask generated with RELION with a soft edge of three pixels. Two classes were set for the 3D classification of these signal-subtracted particles, run without alignments and with the regularization parameter *T* = 50 (61). This resulted in a major class of 15,754 particles that showed improved continuity in the compound **1** cryo-EM density (Fig. S3). The signal-subtracted particle of these 15,754 particles was reverted to the full particle, and a final solvent-flattened FSC 3D refinement was performed that resulted in a 3.4 Å reconstruction (FSC = 0.143) on postprocessing, with a mask generated from the model using UCSF Chimera-1.13 with a low-pass filter of 15 Å and soft edges of five pixels. The map was sharpened according to its local resolution to improve the definition of the compound **1** cryo-EM density, using MonoRes (62) and LocalDeblur (63) in the Scipion-1.2.1 package (64). The classification scheme and local resolution are shown in Figs. S2 and S4.

### Model building, refinement, and validation

The final map was compared against the active (EMDB 11377) and deactive (EMDB 11810) maps low-pass filtered to 3.4 Å. Using the UCSF Chimera v.1.13.1 ‘Fit in Map’ tool, the correlation values for the deactive and active model maps were 0.81 and 0.97 respectively, suggesting the compound **1**-bound complex I is in the active state (in agreement with local features). The active state model of the piericidin A-bound mouse complex I (PDB 6ZTQ) was used as the initial model (29), which was fit into the map using the UCSF Chimera v.1.13.1 ‘Fit in Map’ tool, before removal of the piericidin A molecule and local remodeling using Coot v.0.8.9.1 and Coot v.0.9-pre (65). The model was used to generate a difference map with *phenix.real_space_diff_map* using the locally sharpened map as the reference map for exploration of possible bound compound **1** molecules, using the *hide dust* tool in Chimera X (66) with box dimension of 10 and threshold of 6.7.

For compound **1** and similar compounds, the initial ligand was generated from the isomeric SMILES of the canonical acetogenin bullatacin, obtained from PubChem (67), using the SMILES → 2D tool available in Coot. The molecule was edited using the ligand-building software Lidia, also available in Coot, to match the structure of the compound. The ligand PDB was exported, and CIF file was generated using the eLBOW tool (68). The compound **1** ligand was placed into the model and locally remodeled using the locally sharpened map in Coot. The same method was used to derive the other acetogenin ortholog compounds and restraint files used in the comparative analysis; as this did not include refinement of the surrounding residues, potential contact points were inspected manually in Coot and interactions defined using ChimeraX (66). The log*P* of compound 1 (ChemSpider ID: 23,157,492) was calculated using ChemSpider (http://www.chemspider.com).

Iterations of real-space refinement using PHENIX v.1.19 to 4122 (69) with secondary structure restraints and Coot local remodeling were performed to improve model statistics. Final model statistics were generated in PHENIX using MolProbity (70) and EMRinger (71), as well as *Q*-score analysis (72) using the UCSF Chimera v.1.13.1 MapQ plugin. Model–map correlations were calculated using the CC_mask_ tool (45) for the various poses of the compounds tested.

The continuous surface of the compound **1**/ubiquinone-binding channels were predicted with the online tool CASTp, which uses the topography of the proteins from the input PDB files (73). The default probe size of 1.4 Å was used. The cavity surface was generated as an object using the CASTpyMOL v3.1 plugin for PyMol (http://www.pymol.org/pymol) and visualized in ChimeraX (66).

## Data availability

The structural accession codes for the map and model are EMD-13611 (https://www.ebi.ac.uk/emdb/) and 7PSA (https://www.rcsb.org/), respectively. The associated raw cryo-EM micrographs are available at the Electron Microscopy Public Image Archive (EMPIAR) entry EMPIAR-10927.

## Supporting information

This article contains supporting information (8, 63, 72).

## Conflict of interest

The authors declare that they have no conflicts of interest with the contents of this article.
