# Supplementary material for: Cryo-electron microscopy reveals how acetogenins inhibit mitochondrial respiratory complex I
Source: J Biol Chem. 2022 Jan 19;298(3):101602. doi: 10.1016/j.jbc.2022.101602 (PMC8861642; doi:10.1016/j.jbc.2022.101602)
Supplement: Supplemental Figures S1–S7, Tables S1 and S2 [file mmc1.docx]

**Table S1. Cryo-EM data collection, refinement and validation statistics.**

|  | Acetogenin-bound mouse complex I  (EMD-13611, PDB 7PSA) |
| --- | --- |
| **Data collection and processing** |  |
| Magnification | 130,000× |
| Voltage (kV) | 300 |
| Electron exposure (e^–^/Å^2^) | 51 |
| Defocus range (μm) | -1.5 to -2.7 |
| Pixel size (Å) | 1.043 |
| Symmetry imposed | *C*1 |
| Initial particle images (no.) | 114,209 |
| Final particle images (no.) | 15,754 |
| Map resolution (Å) | 3.4 (4.0) |
| FSC threshold | 0.143 (0.5) |
| Map resolution range (Å) | 3.0–15.0 |
| **Refinement** |  |
| Initial model used | 6ZTQ |
| Model resolution (Å) | 3.3 |
| FSC threshold | 0.5 |
| Map sharpening B factor (Å^2^) | -84 |
| Model composition |  |
| Non-hydrogen atoms | 67,082 |
| Protein residues | 8181 |
| Ligands | 36 |
| B factors (Å^2^) |  |
| Protein | 58.23 |
| Ligand | 59.92 |
| R.m.s. deviations |  |
| Bond lengths (Å) | 0.005 |
| Bond angles (°) | 0.672 |
| **Validation** |  |
| MolProbity score | 1.82 |
| Clashscore | 6.97 |
| Poor rotamers (%) | 0.01 |
| Ramachandran plot |  |
| Favored (%) | 93.23 |
| Allowed (%) | 6.77 |
| Disallowed (%) | 0.00 |
| Rama-Z (RMSD) |  |
| whole (N = 8076) | -1.05 (0.09) |
| helix (N = 4096) | -0.09 (0.08) |
| sheet (N = 373) | -1.81 (0.27) |
| loop (N = 3607) | -1.19 (0.10) |
| Twisted proline/general | 0/0 |
| EMRinger score | 3.02 |
| Model *Q*-score (expected*) | 0.569 (0.516) |

*Based on projections from resolution reference models (72).

**Table S2. Build summary of subunits in the compound 1-bound mouse complex I model.**

| Subunit  Human/bovine  nomenclature | Chain | Total residues* | Residues  modelled (%) | Residues without side  chains | Modelled with side chains (%) | *Q*-score^†^ |
| --- | --- | --- | --- | --- | --- | --- |
| NDUFS1/  75 kDa | G | 704  (1–704) | 98  (6–693) | 0 | 100 | 0.58 |
| NDUFV1/  51 kDa | F | 444  (1–444) | 96  (9–436) | 0 | 100 | 0.56 |
| NDUFS2/  49 kDa | D | 430  (1–430) | 100  (1–430) | 0 | 100 | 0.59 |
| NDUFS3/  30 kDa | C | 228  (1–228) | 91  (7–213) | 0 | 100 | 0.61 |
| NDUFV2/  24 kDa | E | 217  (1–217) | 98  (5–216) | 0 | 100 | 0.55 |
| NDUFS8/  TYKY | I | 178  (1–178) | 100  (1–178) | 0 | 100 | 0.59 |
| NDUFS7/  PSST | B | 189  (1–189) | 83  (34–189) | 0 | 100 | 0.60 |
| NU1M/  ND1 | H | 318  (1–318) | 100  (1–318) | 0 | 100 | 0.58 |
| NU2M/  ND2 | N | 345  (1–345) | >99  (1–344) | 0 | 100 | 0.57 |
| NU3M/  ND3 | A | 115  (1–115) | 100  (1–115) | 0 | 100 | 0.58 |
| NU4M/  ND4 | M | 459  (1–459) | 100  (1–459) | 0 | 100 | 0.58 |
| NU5M/  ND5 | L | 607  (1–607) | >99  (1–606) | 0 | 100 | 0.56 |
| NU6M/  ND6 | J | 172  (1–172) | 99  (1–171) | 0 | 100 | 0.54 |
| NULM/  ND4L | K | 98  (1–98) | 100  (1–98) | 0 | 100 | 0.58 |
| NDUFA9/  39 kDa | P | 342  (1–342) | 100  (1–342) | 0 | 100 | 0.59 |
| NDUFA5/  B13 | V | 115  (1–115) | 99  (2–115) | 0 | 100 | 0.55 |
| NDUFS6/  13 kDa | R | 96  (1–96) | 98  (1–94) | 0 | 100 | 0.62 |
| NDUFS4/  18 kDa/AQDQ | Q | 133  (1–133) | 94  (9–133) | 0 | 100 | 0.60 |
| NDUFA7/  B14.5a | r | 112  (1–112) | 88  (1–77, 90–112) | 0 | 100 | 0.57 |
| NDUFA12/  B17.2 | q | 145  (1–145) | 99  (1–144) | 0 | 100 | 0.59 |
| NDUFA6/  B14 | W | 130  (1–130) | 88  (17–130) | 0 | 100 | 0.59 |
| NDUFAB1/  SDAP | T | 88  (1–88) | 80  (7–82) | 0 | 100 | 0.49 |
| NDUFA2/  B8 | S | 98  (1–98) | 82  (13–95) | 0 | 100 | 0.56 |
| NDUFA8/ PGIV | X | 171  (1–171) | 100  (1–171) | 0 | 100 | 0.57 |
| NDUFA11/  B14.7 | Y | 140  (1–140) | 100  (1–140) | 0 | 100 | 0.54 |
| NDUFA13/  B16.6 | Z | 143  (1–143) | 99  (3–143) | 0 | 100 | 0.56 |
| NDUFS5/  15 kDa/PFFD | e | 105  (1–105) | 100  (1–105) | 0 | 100 | 0.56 |
| NDUFA10/  42 kDa | O | 320  (1–320) | 100  (1–320) | 0 | 100 | 0.58 |
| NDUFA3/  B9 | b | 83  (1–83) | 96  (4–83) | 0 | 100 | 0.56 |
| NDUFA1/  MWFE | a | 70  (1–70) | 97  (1–68) | 0 | 100 | 0.58 |
| NDUFC2/  B14.5b | d | 120  (1–120) | 100  (1–120) | 0 | 100 | 0.57 |
| NDUFB11/  ESSS | g | 122  (1–122) | 83  (21–121) | 0 | 100 | 0.55 |
| NDUFB8/  ASHI | l | 157  (1–157) | 98  (3–156) | 0 | 100 | 0.57 |
| NDUFB5/  SGDH | h | 143  (1–143) | 97  (6–143) | 0 | 100 | 0.58 |
| NDUFB3/  B12 | k | 103  (1–103) | 73  (19–93) | 0 | 100 | 0.51 |
| NDUFB4/  B15 | m | 128  (1–128) | 98  (3–128) | 0 | 100 | 0.53 |
| NDUFB7/  B18 | o | 136  (1–136) | 82  (2–112) | 0 | 100 | 0.51 |
| NDUFAB1/  SDAP | U | 88  (1–88) | 98  (3–88) | 0 | 100 | 0.53 |
| NDUFB10/  PDSW | p | 175  (1–175) | 97  (4–172) | 0 | 100 | 0.53 |
| NDUFB9/  B22 | n | 178  (1–178) | 99  (1–177) | 0 | 100 | 0.56 |
| NDUFB2/  AGGG | j | 72  (1–72) | 86  (7–68) | 0 | 100 | 0.51 |
| NDUFB6/  B17 | i | 127  (1–127) | 73  (1–36, 66–123­­) | 0 | 100 | 0.55 |
| NDUFV3/  10 kDa | s | 69  (1–69) | 59  (28–68) | 0 | 100 | 0.58 |
| NDUFC1/  KFYI | c | 49  (1–49) | 99  (1–48­­) | 0 | 100 | 0.53 |
| NDUFB1/  MNLL | f | 56  (1–56) | 95  (4–56) | 0 | 100 | 0.53 |

*Based on values in (8). †(72)


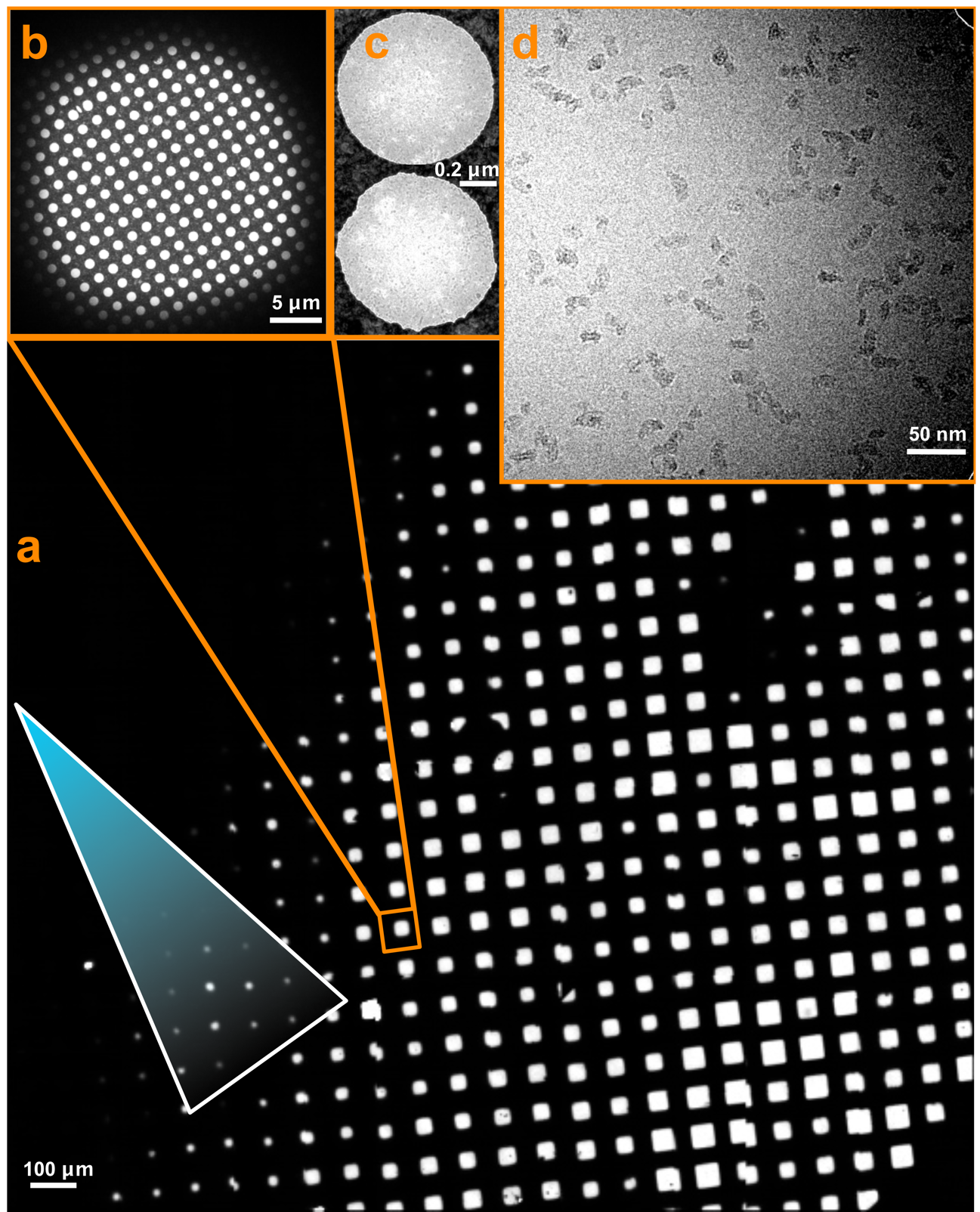


**Figure S1. The grid used for data collection.** The grid was imaged using a 300 keV Titan KriosTM EM microscope at eBIC (Electron Bio-Imaging Centre) at Diamond Light Source (Harwell Science and Innovation Campus, Didcot, UK). (**a**) The atlas (grid overview) with orange box insets describing (**b**) a representative grid square, (**c**) foil-hole overview examples, and (**d**) example hole particle distribution. The centres of the holes contained thin ice and so images were shot slightly off centre to avoid this. The triangle gradient indicates the ice thickness across the grid, with thicker ice found at the upper left of the atlas. Relatively thick ice provided better complex I particle distribution.


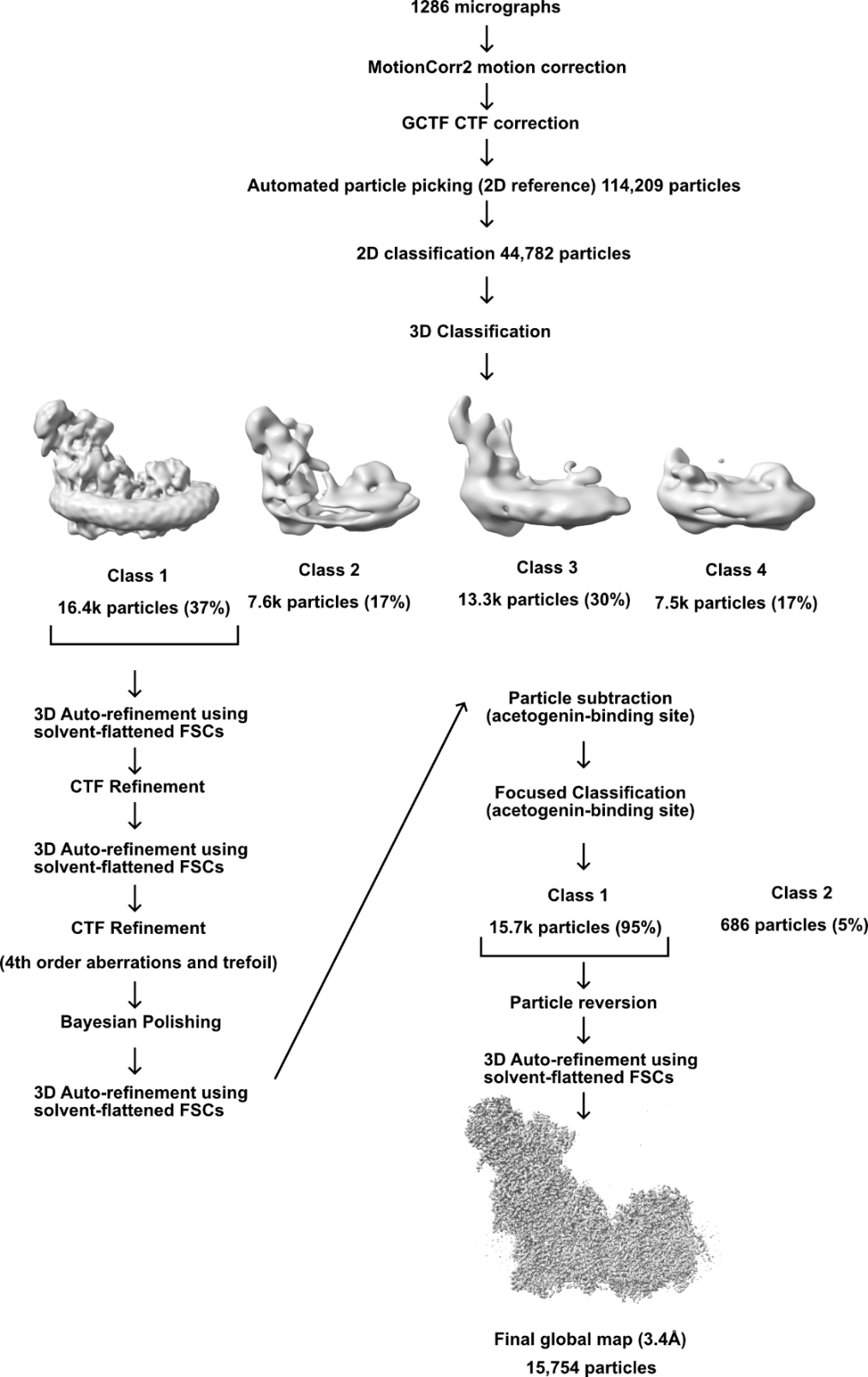


**Figure S2. The final classification scheme used in RELION-3.1.** RELION-3.1 introduced per-particle CTF corrections accounting for higher order aberrations. The focused classification was performed on polished particles and the final global reconstruction resulted in 3.4 Å resolution from 15,754 particles. Initial 2D classification removed non-complex I particles. Classes 2–4 of the 3D classification represent aggregated/broken complexes similar to that observed previously (8). No deactive state was classified from the 16.4k particles even with higher angular sampling.


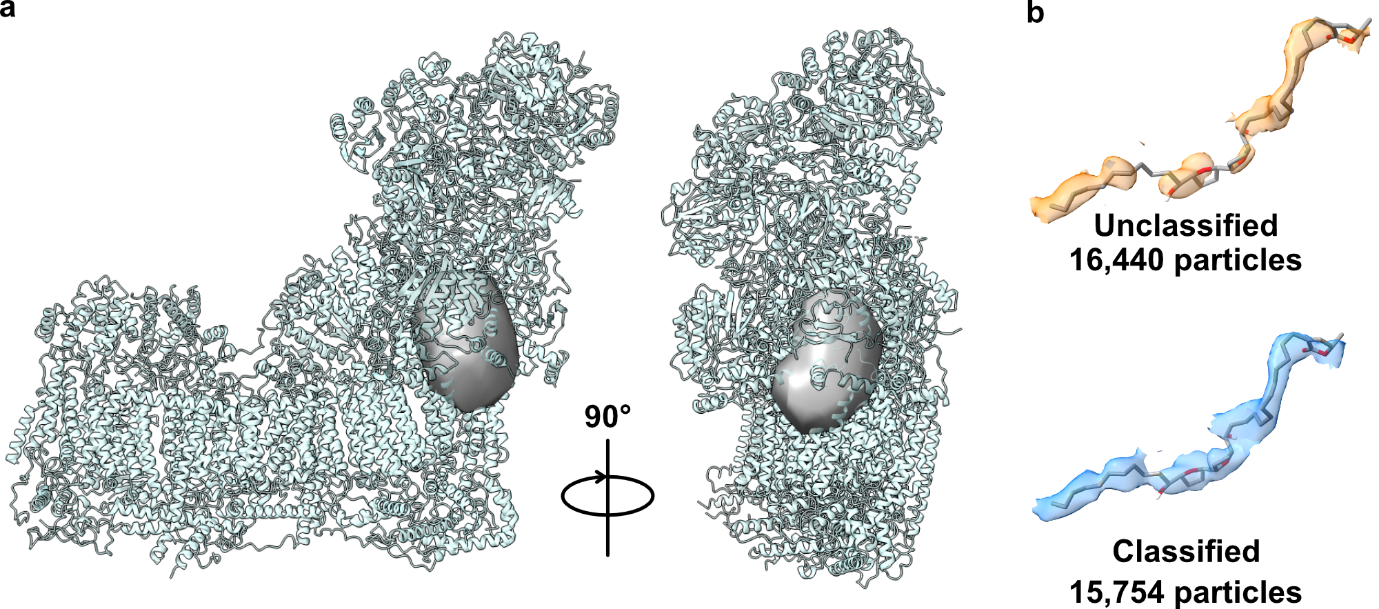


**Figure S3. Focused 3D classification around the compound 1-binding site.** A mask for the focused 3D classification was generated from a 7 Å radius around the compound **1**-binding site that was low-pass filtered to 15 Å. (**a**) The mask volume used for the classification without alignments is shown in grey surface on a model of the reconstruction. (**b**) The comparison of the compound **1**-binding site of the final reconstructions before and after the focused 3D classification. Both maps are locally sharpened with LocalDeblur (63). The major class after the classification shows improvement in compound **1** cryo-EM density, despite the fewer particles, suggesting the removed particles contain empty binding sites or are residual junk particles not classified out in the initial 3D classification stage. ChimeraX threshold = 0.0481.


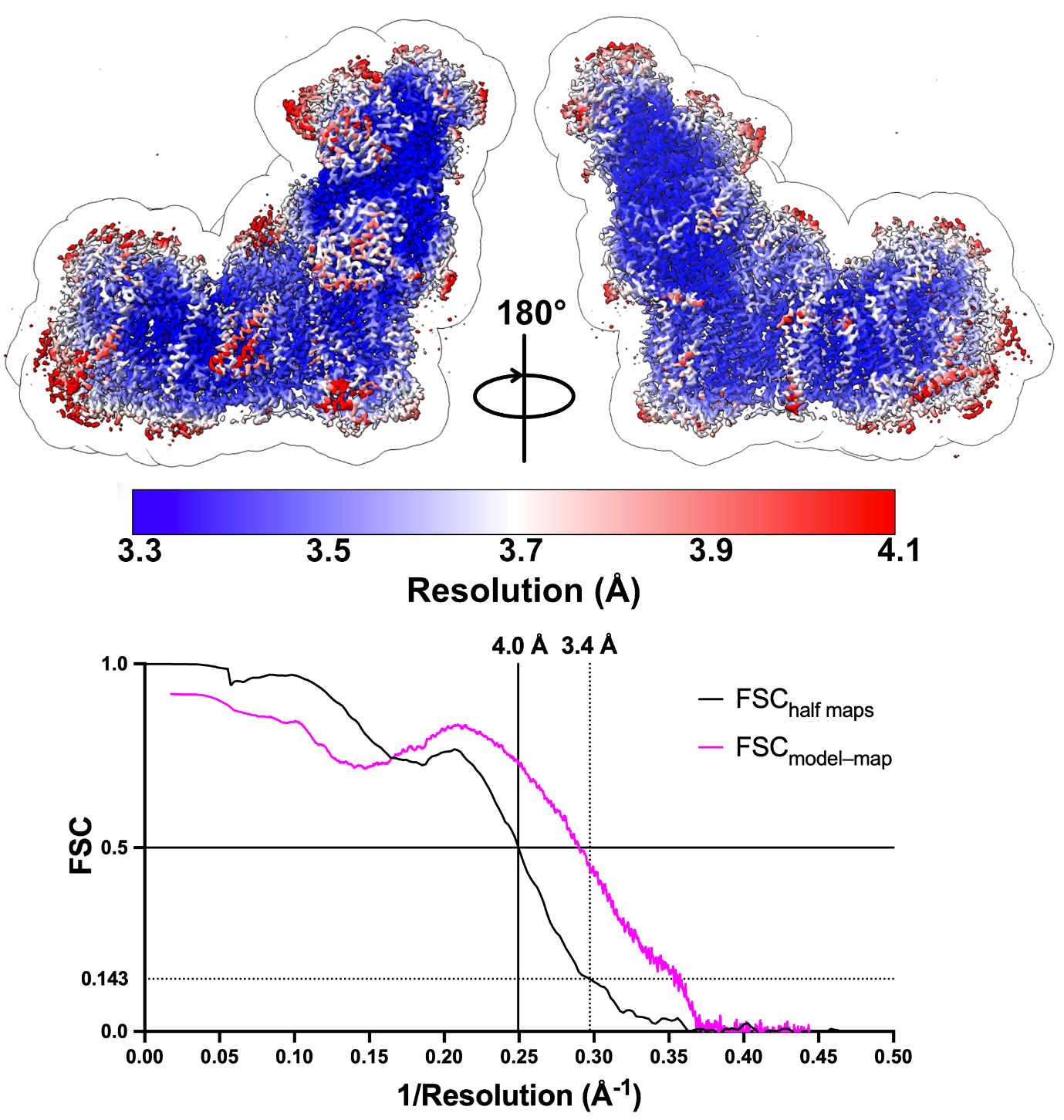


**Figure S4. The local resolution of the final 3.4 Å global map output in RELION-3.1.** The distal peripheries show the worst local resolution. The black outline shows the outline of the mask used for the final post-processing in RELION-3.1. The local resolution is indicated by the colour bar. The resolution estimates from the masked Fourier shell correlation curves, FSC_half maps_, are 3.4 Å for the global at FSC = 0.143 (dotted lines) and 4.0 Å at FSC = 0.5 (solid lines). Model–map FSC curve (FSC_model–map_) is in magenta.


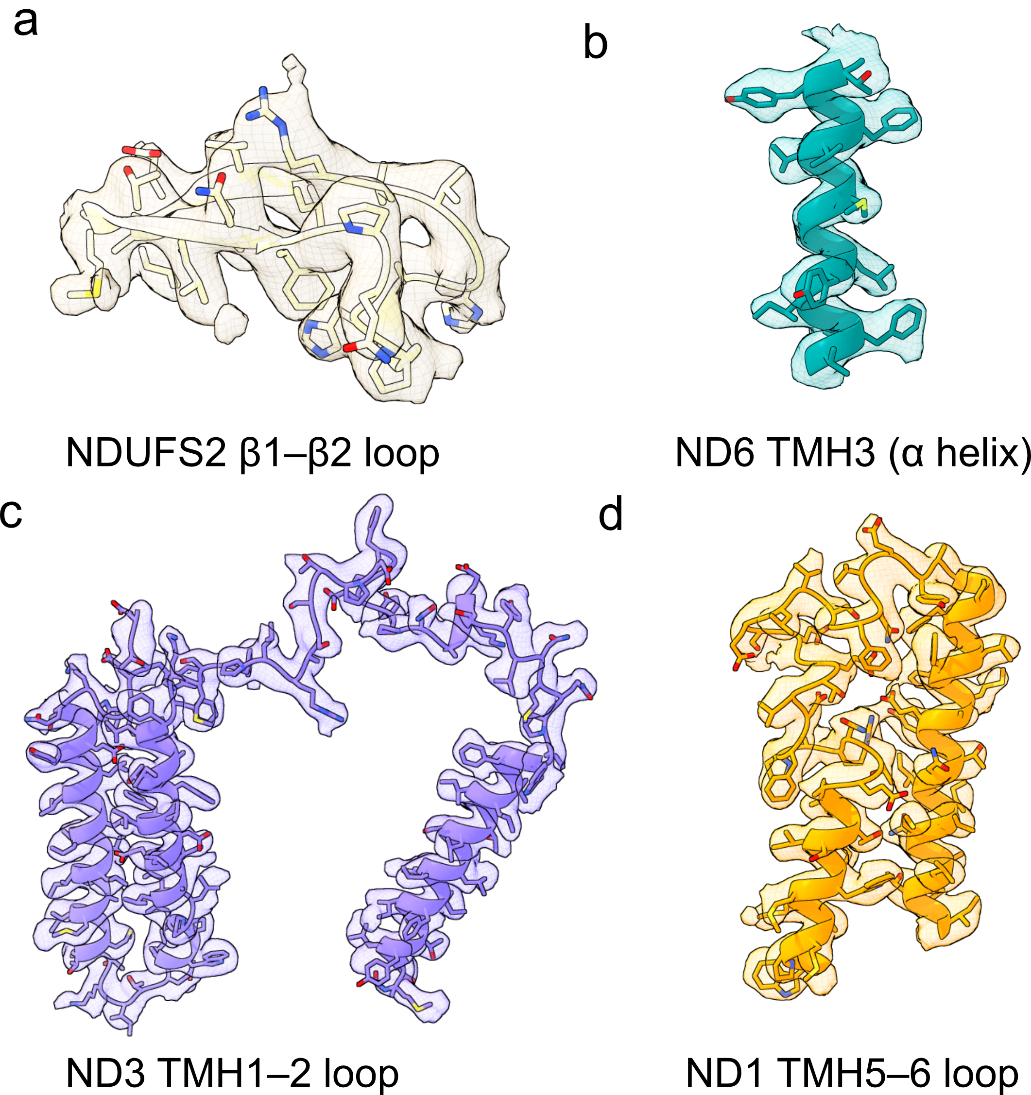


**Figure S5. Structural elements relevant to the active/deactive enzyme status.** (**a**) The β1–β2 hairpin loop of NDUFS2 that forms part of the binding channel and also contains the catalytic histidine. (**b**) TMH3 of ND6 in the α-helical conformation, only observed in the active state. (**c**) The ND3 subunit with the central loop between TMHs 1 and 2 spanning across the binding site at the hydrophilic/hydrophobic domain interface. (**d**) The acidic loop of ND1 between TMHs 5 and 6 that forms the lower part of the binding channel. Cryo-EM density is shown as a semi-transparent surface. (ChimeraX threshold 0.03 for **a**–**d**).


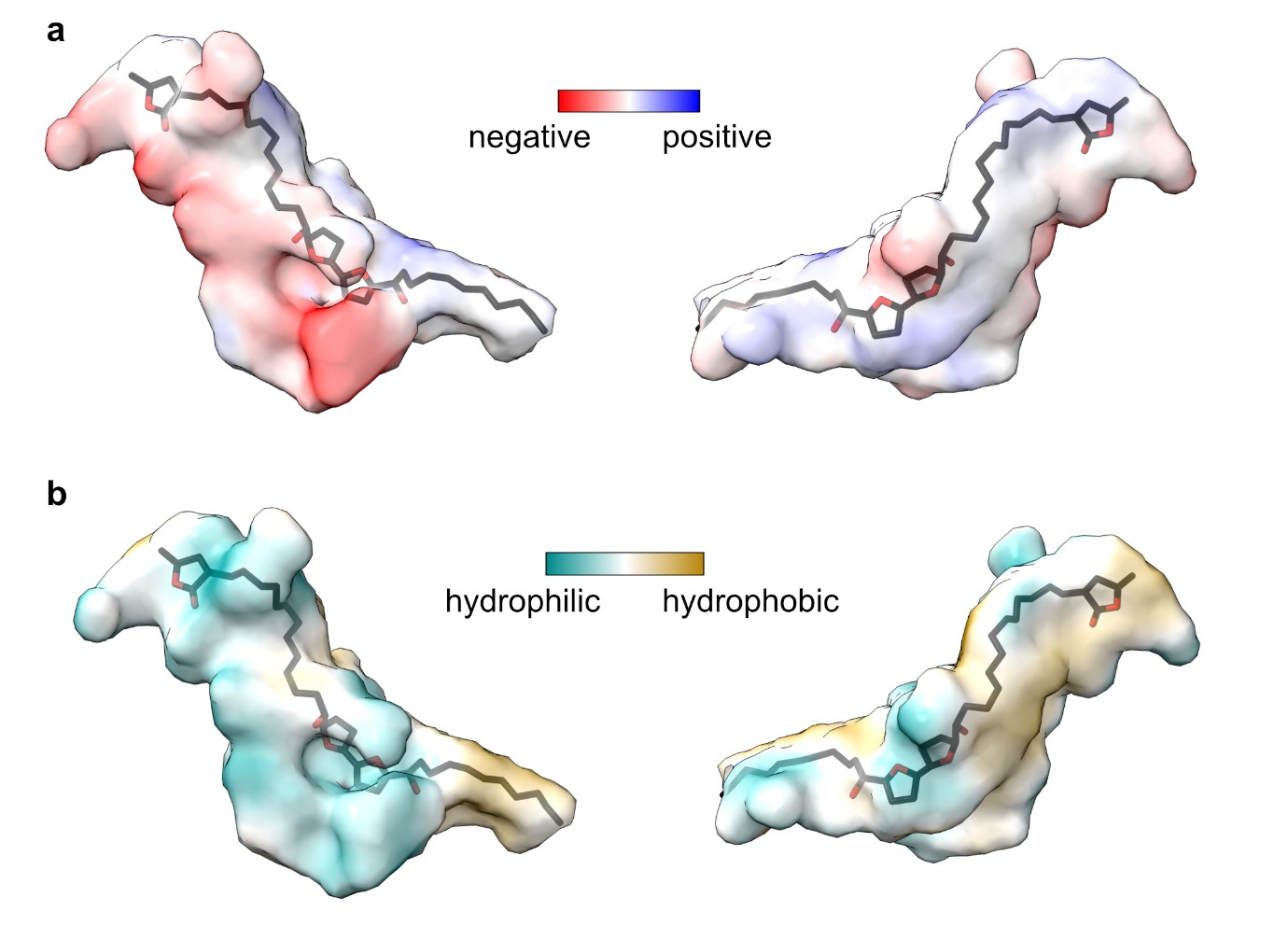


**Figure S6. The chemical properties of the solvent-probe cavity of the compound 1-binding site.** (**a**) The Coulombic electrostatic potential of the protein surrounding the compound **1**-binding site. Default parameters were used in ChimeraX to calculate the values, with the colours representing values determined as indicated by the key (red, negative; white, neutral; blue, positive). (**b**) The lipophilicity potential of the protein surrounding the compound **1**-binding site. Default parameters were used in ChimeraX to calculate the values, with the colours representing values determined as indicated by the key (cyan, hydrophilic; white, neutral; gold, hydrophobic).


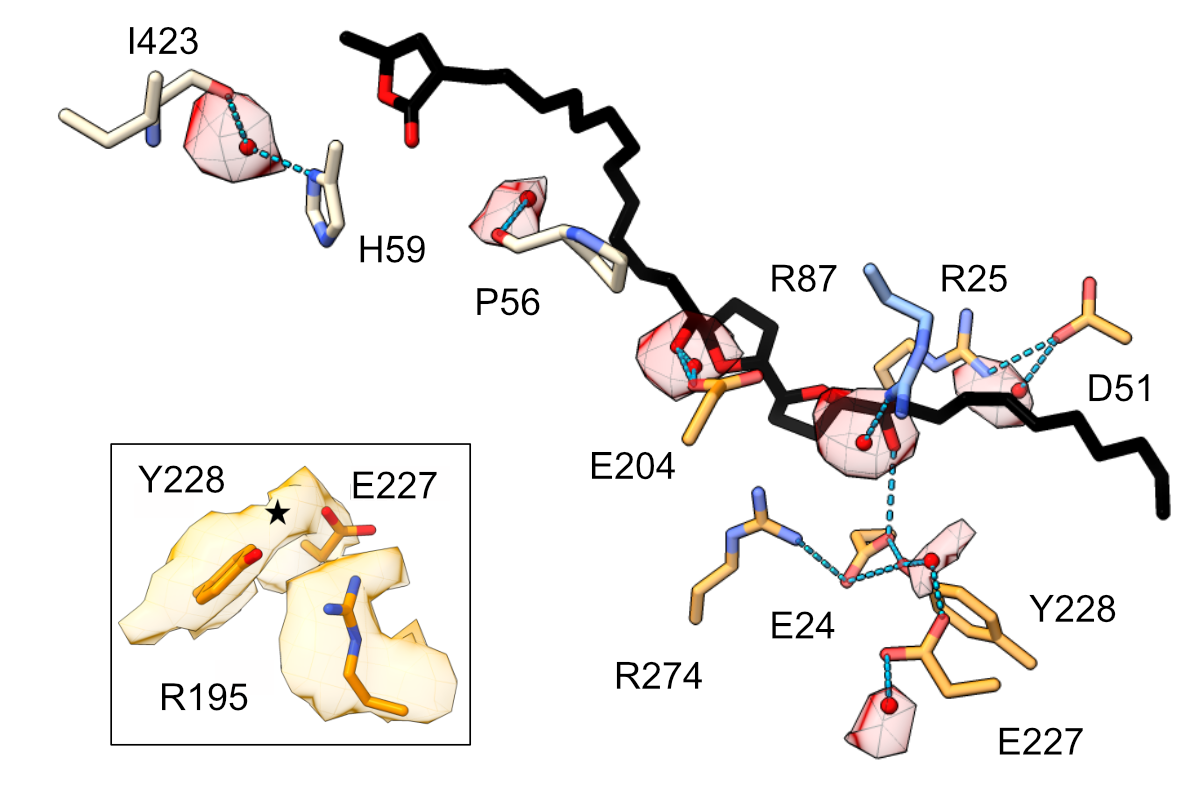


**Figure S7. Possible hydration around the bound acetogenin compound 1.** Water molecules (red spheres) are placed in possible cryo-EM density spots of the locally sharpened map (carved from a 2 Å distance of the modelled waters at a ChimeraX threshold of 0.03) that surround the bound compound **1** (black). Hydrogen bonds are shown in cyan dashed lines. The C15 hydroxyl group forms a hydrogen bond with a water molecule that in turn interacts with ND1-E204. A possible water sits nearby the C24 hydroxyl, bonded to NDUFS7-R87. NDUFS2 residues are shown in wheat, NDUFS7 in cyan, and ND1 in orange. A possible water molecule between E227 and Y228 mentioned in the main text is also shown here, with inset showing the cryo-EM density for this region (black star indicates possible water position).
